# Towards a “canonical” agranular cortical microcircuit

**DOI:** 10.3389/fnana.2014.00165

**Published:** 2015-01-14

**Authors:** Sarah F. Beul, Claus C. Hilgetag

**Affiliations:** ^1^Department of Computational Neuroscience, University Medical Center Hamburg-EppendorfHamburg, Germany; ^2^Department of Health Sciences, Boston University, BostonMA, USA

**Keywords:** cytoarchitecture, intrinsic circuitry, interlaminar connectivity, striate cortex, structural variation

## Abstract

Based on regularities in the intrinsic microcircuitry of cortical areas, variants of a “canonical” cortical microcircuit have been proposed and widely adopted, particularly in computational neuroscience and neuroinformatics. However, this circuit is founded on striate cortex, which manifests perhaps the most extreme instance of cortical organization, in terms of a very high density of cells in highly differentiated cortical layers. Most other cortical regions have a less well differentiated architecture, stretching in gradients from the very dense eulaminate primary cortical areas to the other extreme of dysgranular and agranular areas of low density and poor laminar differentiation. It is unlikely for the patterns of inter- and intra-laminar connections to be uniform in spite of strong variations of their structural substrate. This assumption is corroborated by reports of divergence in intrinsic circuitry across the cortex. Consequently, it remains an important goal to define local microcircuits for a variety of cortical types, in particular, agranular cortical regions. As a counterpoint to the striate microcircuit, which may be anchored in an exceptional cytoarchitecture, we here outline a tentative microcircuit for agranular cortex. The circuit is based on a synthesis of the available literature on the local microcircuitry in agranular cortical areas of the rodent brain, investigated by anatomical and electrophysiological approaches. A central observation of these investigations is a weakening of interlaminar inhibition as cortical cytoarchitecture becomes less distinctive. Thus, our study of agranular microcircuitry revealed deviations from the well-known “canonical” microcircuit established for striate cortex, suggesting variations in the intrinsic circuitry across the cortex that may be functionally relevant.

## Introduction

The cerebral cortex is arguably one of the most complex physical systems. Untangling the intricate relations of the myriad elements of the gray matter is one of the formidable challenges of science, as already pronounced by Santiago Ramon y Cajal:

“Devotion to the cerebral hemispheres, enigma of enigmas, was old in me…the supreme cunning of the structure of the gray matter is so intricate that it defies and will continue to defy for many centuries the obstinate curiosity of investigators. That apparent disorder of the cerebral jungle, so different from the regularity and symmetry of the spinal cord and of the cerebellum, conceals a profound organization of the utmost subtlety which is at present inaccessible.” (Cajal, 1937)

Decades later, the picture has become more refined, but a comprehensive understanding of the cortical microarchitecture still remains a fundamental scientific challenge. A crucial step was the recognition that the cerebral cortex is locally structured into horizontal compartments (“layers”) as well as vertical units (“columns”) which both may be of functional relevance. Traditionally, the isocortex has been characterized in the context of a six-layered scheme (Brodmann, 1909; Vogt, 1910; von Economo, 2009), as opposed to three-layered allocortex. This scheme is, however, subject to substantial variation in the relative prominence of layers and disrupted in a considerable number of cortical areas. Nonetheless, and in spite of his acknowledgment that “the distinction of six layers can be both arbitrary and conventional” (von Economo, 2009), already von Economo himself asserted that “on practical grounds, we retain the six-layer division” (von Economo, 2009). Indeed, the simplified concept of a uniformly six-layered isocortex has prevailed (Zilles and Amunts, 2012) and become generally accepted.

**Figure 1:**
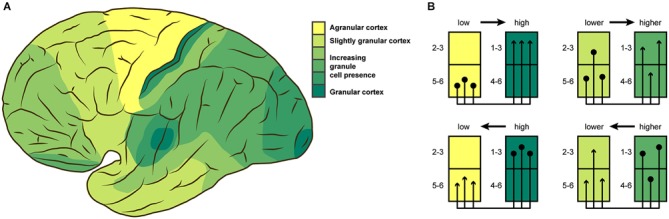
**(A)** Cytoarchitectonic differentiation varies across the cortex. This lateral view of the human brain shows broad variations in granule cell presence as described by von Economo (2009). **(B)** Laminar origin and termination patterns of extrinsic cortico-cortical connections vary according to the relative architectonic differentiation of the connected areas. Projection origins (terminations) shift from infragranular to supragranular layers, as the source (target) area becomes more strongly differentiated. This rule results in unilaminar profiles for projections between areas that are unequal in their differentiation, and multilaminar profiles for areas with more similar differentiation. **(A)** adapted from von Economo (2009), **(B)** adapted from Barbas and Rempel-Clower (1997).

The radial organization of the cortex became a subject of interest when vertical columns spanning all cortical layers were proposed to exist (Lorente de Nó, 1949; Mountcastle, 1957), with uniform columns repeating across the cortex to form an intermediate-level neural substrate for information processing. Within these columns, connectivity across cortical layers appeared stereotypical (Szentagothai, 1978; Gilbert and Wiesel, 1983). While there is still considerable debate about the existence, precise definition and the extent of heterogeneity in the cellular composition of cortical columns (Rakic, 2008; da Costa and Martin, 2010; Rockland, 2010; Smith, 2010a,b,c,d; Carlo and Stevens, 2013; Herculano-Houzel et al., 2013), the concept of radial cortical organization was later extended to the notion of a “canonical” microcircuit (Douglas et al., 1989; Douglas and Martin, 1991, 2004), as a generic template of intrinsic cortical circuitry. The computations performed by such a fundamental neuronal circuit are thought to be prescribed by the intrinsic circuitry within a cortical column, with functional specificity added by patterns of axonal inputs and outputs to and from the column. Substantial work has been devoted to the computational performance and theoretical properties of the “canonical” microcircuit (e.g., Douglas et al., 1989, 1995; Haeusler and Maass, 2007; George and Hawkins, 2009; Haeusler et al., 2009; Wagatsuma et al., 2011; Bastos et al., 2012; Habenschuss et al., 2013). In the primate prefrontal cortex, the “canonical” microcircuit was shown to be subject to modifications from the striate circuit (Heinzle et al., 2007; Godlove et al., 2014). More generally, abundant data is available on variants of intrinsic connectivity in cortical regions such as prefrontal cortex (Melchitzky et al., 2001), somatosensory cortex (Lübke and Feldmeyer, 2007; Petersen, 2007; Lefort et al., 2009; Feldmeyer et al., 2013) or auditory cortex (Barbour and Callaway, 2008; Oviedo et al., 2010; Watkins et al., 2014). Nonetheless, the notion of a “canonical” microcircuit, which has gained popularity especially in the computational neuroscience community and has also inspired neuroengineering solutions (e.g., Merolla et al., 2014), is still largely based on work in one particular cortical area, striate cortex. Moreover, much of this work has concentrated on the cat and non-human primate brain (Douglas and Martin, 2007a). Striate cortex is not only special in the amount of probing it has undergone, but is also exceptional in its cytoarchitectonic differentiation. Striate cortex is the cortical region with the highest neuron density, sporting numbers substantially higher than the remainder of the cortex (Schüz and Palm, 1989; Collins et al., 2010; Cahalane et al., 2012; Herculano-Houzel et al., 2013). The number of (sub)layers that can be identified is also higher in striate cortex than in other regions of the cortex. Instead of all parts of the cortex being uniformly differentiated, cytoarchitectonic differentiation changes gradually across the cortex (Sanides, 1970; von Economo, 2009; Zilles and Amunts, 2012), as illustrated in Figure 1A for the human brain. The spectrum of differentiation ranges from striate cortex, the most clearly eulaminate area, to agranular areas that lack the inner granular layer (L4), and have few identifiable sublayers as well as very low neuron density. In between these two extremes, one can find areas that are still eulaminate, but without the remarkable clarity of differentiation or dense packing of neurons found in striate cortex, such as prestriate cortex, as well as dysgranular areas with a lower density of neurons, a dissolving inner granular layer and fewer identifiable sublayers. Quantitative differences in many aspects of the structural organization of cortical tissue have been extensively demonstrated (e.g., Beaulieu and Colonnier, 1989; Defelipe et al., 1999; Dombrowski et al., 2001; Yáñez et al., 2005; Collins et al., 2010).

The variation in local cortical structure needs to be taken into account when describing a “canonical” microcircuit, because it is unlikely for the patterns of inter- and intra-laminar connections to be uniform in spite of strong variations of their structural substrate. Indeed, experimental results, for example from rodent barrel cortex, demonstrate that intrinsic connectivity is not uniform across the cortex (Sato et al., 2008; Meyer et al., 2013; Reyes-Puerta et al., 2014). Heterogeneity in cytoarchitectonic differentiation has been shown to have consequences for other aspects of structural connectivity in the brain. The laminar patterns of extrinsic connections which link brain regions along white matter pathways are strongly associated with the relative cytoarchitectonic differentiation of the connected areas (Barbas, 1986; Barbas and Rempel-Clower, 1997; Medalla and Barbas, 2006; Hilgetag and Grant, 2010; Beul et al., 2014). The stereotypic laminar patterns that have been found in non-human primate and cat cortex (Figure 1B) show distinctly infra- and supragranular origins and terminations for projections between areas of weak differentiation and areas of strong differentiation, while these patterns change gradually towards multilaminar origin and termination profiles as the difference in differentiation between the connected areas becomes less pronounced.

Since the variation of cytoarchitectonic differentiation is an aspect of cortical organization that is insufficiently considered in discussions of intrinsic circuitry, we here want to raise awareness of the importance of architectonic differences, by providing a first approximation of general features of intrinsic circuitry in agranular regions of the cerebral cortex. We do this by assimilating information from the available literature on inter- and intralaminar connectivity in the agranular frontal cortex of the rodent brain, in order to present a tentative model of intrinsic circuitry in cortical regions on the opposite end of the differentiation spectrum than has previously been predominantly considered for such models. This variation is crucial for applying insights gained from such model circuits in a realistic way, for example in the biological grounding of *in silico* experiments (e.g., Merolla et al., 2014).

In the following review, we briefly introduce current accounts of the “canonical” microcircuit, and then highlight a report of experimental results that reveal variation in interlaminar inhibition across cortical regions of distinct cytoarchitecture (Kätzel et al., 2011). Subsequently, we present the result of the literature survey we performed regarding data that can shed light on the intrinsic microcircuitry in agranular cortex. We chose to concentrate on the rodent brain, capitalizing on the relative abundance of experimental data available for this popular animal model. In comparison, fewer studies report on intrinsic circuitry in non-human primates, and only a small proportion of those considered agranular cortical regions, which are relatively infrequent in the primate brain. By focusing on the rodent brain, we can therefore provide a more detailed sketch of the intrinsic circuitry in agranular cortex without having to incorporate data across a wide range of species, which would have been a more uncertain approach.

## Intrinsic circuitry in granular cortex

Over the last decades, general features of intrinsic circuitry in striate cortex have emerged from studies in the cat and non-human primate. Connections are proposed to form a processing loop across cortical layers, where recurrent excitation and inhibition are interlinked, which leads to amplification of inputs into the cortical column and appropriate modulation of the ensuing activity (Markram et al., 2004; Douglas and Martin, 2004, 2007a; Bannister, 2005; Lübke and Feldmeyer, 2007). To probe the local microcircuitry, diverse experimental methods with different degrees of sensitivity and reliability have been used. Two investigations that supplied the most comprehensive data on cat striate cortex employed electrophysiological and morphological approaches, respectively. Thomson et al. (2002) used dual intracellular recordings to characterize synaptic connections across cortical layers. Binzegger et al. (2004) reconstructed the morphology of neurons in striate cortex in three dimensions and estimated the number of synaptic contacts between different cell types. Both data sets have been adapted and used in various studies, for example, in the construction of computational models (e.g., Haeusler and Maass, 2007; Haeusler et al., 2009; Bastos et al., 2012; Du et al., 2012; Potjans and Diesmann, 2014). But even though the same model system, cat striate cortex, was considered across these studies, there currently exists no definite scheme of this area’s intrinsic circuitry. There are, for example, diverging data on whether recurrent excitation occurs between L3 and L5 or between L4 and L3 (cf. Thomson et al., 2002; Thomson and Bannister, 2003 and Binzegger et al., 2004; Douglas and Martin, 2004).

**Figure 2:**
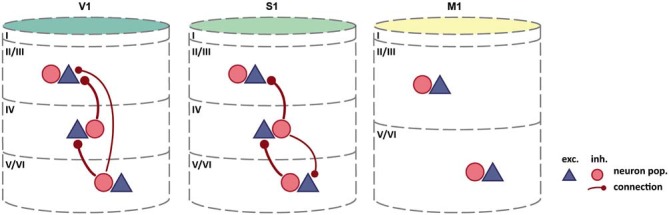
**Interlaminar inhibition varies across mouse cortex**. As cytoarchitectonic differentiation becomes weaker, the abundance of interlaminar inhibitory-to-excitatory connectivity decreases. By contrast, intralaminar connectivity, including intralaminar inhibition, appears relatively unchanged (Intra-laminar connections, which are all-to-all, are not shown). Column colors follow the color coding of cytoarchitectonic differentiation in Figure 1: yellow-weakly differentiated cortex to dark green-strongly differentiated cortex. Adapted by permission from Macmillan Publishers Ltd: Kätzel et al. (2011).

Such discrepancies may be reconciled by future experimental findings. In contrast, reports of differences in interlaminar activation patterns across cortical regions point towards the existence of genuine variations in intrinsic circuitry across the brain. Kätzel et al. (2011) used genetically targeted photostimulation to comprehensively map inhibitory-to-excitatory connectivity in three distinct regions of mouse cortex. They assessed intra- and interlaminar connectivity in striate cortex, primary somatosensory and primary motor cortex. As mentioned before, striate cortex is by far the most differentiated cortical region, even in the rodent brain (Herculano-Houzel et al., 2013), where it is less well differentiated than for example in the primate. Primary somatosensory cortex, although still clearly eulaminate, is already much less dense and comprises fewer distinguishable sublayers, while primary motor cortex is even less cytoarchitectonically differentiated (Collins et al., 2010; Herculano-Houzel et al., 2013). Primary motor cortex thus ranges in the lower end of the differentiation spectrum with dysgranular cortical regions, although it is sometimes classified as agranular (lacking an inner granular layer, L4): see Shipp (2005) and García-Cabezas and Barbas (2014) for an extensive discussion of this issue. Other than probing connectivity in three cortical regions processing different modalities, this study can, therefore, be used to demonstrate potential differences regarding intrinsic circuitry in three areas occupying different positions in the differentiation spectrum. While Kätzel et al. (2011) report relatively uniform patterns of intralaminar inhibition across these three cortical regions, interlaminar inhibitory-to-excitatory connectivity differed substantially (Figure 2). In striate cortex, considerable interlaminar inhibition was observed between all layers (L2/3, L4, L5/6). In primary somatosensory cortex, a similar pattern of interlaminar inhibition was reported, but without inhibition between L2/3 and L5/6. In primary motor cortex, in contrast, no substantial inhibition between L2/3, L4, and L5/6 was evident. Thus, across the three sampled regions, interlaminar inhibitory-to-excitatory connectivity was progressively weaker in less cytoarchitectonically differentiated areas. Interpreting the results this way, we assume that they reflect genuine variation in the presence of interlaminar inhibition, and not the impact of other aspects of structural variation across the studied areas. For example, systematic differences in cellular morphology across the sampled areas could lead to skewed results from applying the same measurement approach to all areas. Nonetheless, these observations support the notion that intrinsic circuitry cannot be uniform in the face of considerable variation of the structural substrate, as is the case in regions of the cerebral cortex with profoundly differing cytoarchitectonic differentiation.

## Tentative intrinsic circuitry of the agranular cortex

**Figure 3:**
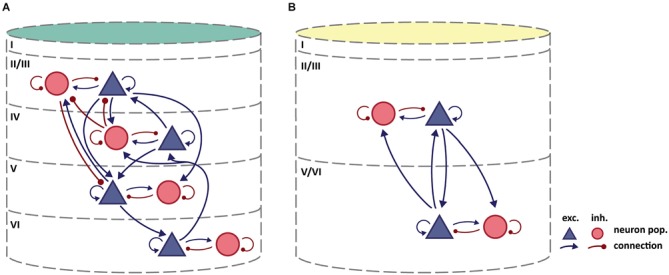
**(A)** Intrinsic circuitry in granular cat striate cortex. Adapted from Potjans and Diesmann (2014) who largely based their diagram on Binzegger et al. (2004). **(B)** Tentative scheme of intrinsic circuitry in agranular rodent frontal cortex. Intralaminar connectivity in agranular cortex is similar to that in granular cortex, but interlaminar connectivity differs. Column colors follow the color coding of cytoarchitectonic differentiation in Figure 1: yellow-weakly differentiated cortex to dark green-strongly differentiated cortex.

Figure 3 summarizes our review of the available literature on intrinsic interlaminar circuitry in the agranular frontal cortex of the rodent brain and puts it in comparison to a recent rendering of the intrinsic circuitry in striate cortex. Excitatory-to-excitatory connections from L2/3 to L5 have clearly been demonstrated in rat agranular frontal cortex by measuring excitatory postsynaptic currents (EPSC) in monosynaptically coupled pyramidal neurons in L5, induced by stimulation in L2/3 (Kang, 1995; Otsuka and Kawaguchi, 2008, 2009, 2011; Hirai et al., 2012). One of these paired recording studies (Otsuka and Kawaguchi, 2009) additionally demonstrated the existence of excitatory-to-inhibitory connections from L2/3 to L5, a finding also reported by Apicella et al. (2012) in mouse motor cortex. The experiments of Hirai et al. (2012) showed that reciprocal connections to the excitatory-to-excitatory connections from L2/3 to L5 exist from L5 pyramidal cells to L2/3 pyramidal cells. This observation is confirmed in medial entorhinal cortex of the rat (van Haeften et al., 2003), which can be considered agranular since its layer IV (“lamina dissecans”) is mainly acellular (Andersen et al., 2007). The microscopy study of van Haeften et al. (2003) traced the processes of pyramidal cells in the deep layers ramifying in superficial layers, and identified the synaptic contacts made by those neurons. The analysis revealed excitatory-to-excitatory, as well as excitatory-to-inhibitory, connections from deep to superficial layers.

Considering the trend of weakening inhibitory-to-excitatory connectivity in cytoarchitectonically less differentiated areas (Kätzel et al., 2011, see above), we consider it likely that there exists no substantial interlaminar inhibition of excitatory neurons in rodent agranular frontal cortex, which is reflected in our tentative circuit diagram. The study by van Haeften et al. (2003) in medial entorhinal cortex, which reports an absence of inhibitory-to-excitatory synapses from deep to superficial layers, supports the same conclusion. Van Haeften et al. furthermore report that only a small percentage of the observed synapses could potentially be classified as inhibitory-to-inhibitory, thus giving little evidence for such a connection from deep to superficial layers. Considering the reciprocal inhibitory-to-inhibitory connection from superficial to deep layers, we could find no studies reporting either on the absence or presence of such a connection. In the circuit diagram (Figure 3), we did not include connections which could only be inferred from exclusively morphological results (e.g., Kawaguchi, 1993, 1995; Kawaguchi and Kubota, 1997; Kubota et al., 2011), since we did not consider data on the spatial spread of axon collaterals sufficiently reliable to demonstrate a functional connection, given that synapse formation has been shown to be highly specific (e.g., Kozloski et al., 2001; Brown and Hestrin, 2009). For these reasons, Figure 3B indicates no inhibitory interlaminar connections, although the validity of this assessment of course remains contingent upon further experimental data.

By contrast, there is abundant evidence for rich intralaminar connectivity including excitatory-to-inhibitory and inhibitory-to-excitatory connections (Kang, 1995; Somogyi et al., 1998; Kawaguchi and Kondo, 2002; Barthó et al., 2004; Otsuka and Kawaguchi, 2009; Fino and Yuste, 2011; Kätzel et al., 2011). Therefore, we assumed a stereotypical pattern of connectivity within deep and superficial layers as illustrated in Figure 3B.

The intrinsic circuitry we have sketched here thus comprises interlaminar excitatory connections that connect neuronal populations from both upper and lower layers to excitatory as well as inhibitory neuron populations in the complementary cortical layers. Within upper and lower layers, intralaminar connections reciprocally connect excitatory and inhibitory neuron populations. This intrinsic interlaminar circuitry is strikingly similar to the simplified original circuit diagram for the striate cortex of Douglas et al. (1989), and allows for recurrent excitation and inhibition. These physiological interactions were inferred to underlie essential computational mechanisms in striate cortex (Douglas et al., 1995; Douglas and Martin, 2007b, 2009). The microcircuitry as we sketch it here should accordingly be able to support elemental neural functions, such as the amplification of weak inputs through positive feedback or gain control and signal normalization through negative feedback.

## Discussion

The starting question of this review was whether there exists a generic template of intrinsic microcircuitry in the cortex, despite pronounced regional differences in cytoarchitectonic organization. The answer depends strongly on how broadly the concept of stereotypy is framed (Silberberg et al., 2002), but even for the cortical region studied most intensely in this context, striate cortex, there exists as yet no consensus on a detailed “canonical” microcircuit. Moreover, differences in circuitry have been reported across the cortex, which are consistent with the changes in the structural substrate in which intrinsic connectivity is embedded. In order to account for these structural differences, we propose a tentative circuit diagram for the agranular frontal cortex of the rodent brain, an agranular region which is strikingly opposed to striate cortex in its cytoarchitectonic organization. Our review of the existing literature points to an intrinsic circuit that features excitatory-to-excitatory and excitatory-to-inhibitory connections from upper layers to lower layers, as well as from lower layers to upper layers (Figure 3B), but shows no interlaminar inhibitory-to-inhibitory or inhibitory-to-excitatory connections. This circuit is based on multiple approaches for structural and functional circuit investigation (such as electrophysiological paired recordings using microstimulation, anatomical tracing experiments, or examination of morphological features using light and electron microscopy), with different caveats and varying levels of reliability. Importantly, the information was drawn from studies whose primary goal was not necessarily the characterization of interlaminar circuitry. Our circuit diagram is therefore subject to debate and should be modified in the light of future information. In compiling the circuit diagram, we engaged in some common simplifications regarding the anatomical substrate in which the connections are placed. In studying intrinsic circuitry, distinct sublayers are often collapsed, as for example when layers 5A, 5B and 6 are considered collectively as “infragranular” layers. This treatment may be misleading, since different (sub)layers have been shown to be involved in distinct processing circuits (for example, Lübke and Feldmeyer, 2007). The same caveat holds for the merging of diverse neuron types into the two main classes of inhibitory and excitatory neurons. It discards a wealth of functionally relevant information about morphological and physiological differences between neuron types, as well as about cell type specific connectivity (Kozloski et al., 2001; Silberberg et al., 2002; Thomson and Bannister, 2003; Kampa et al., 2006; Otsuka and Kawaguchi, 2008, 2009, 2011; Brown and Hestrin, 2009; Xu and Callaway, 2009; Apicella et al., 2012; Hirai et al., 2012). Not to disambiguate such significant anatomical features introduces additional uncertainty about the validity of any intrinsic circuit diagram. Moreover, note that a description of general layer-to-layer connectivity within a column, as we propose here, does not necessarily reflect synaptic circuits formed by individual neurons across layers, as, for example, Binzegger et al. (2004) have estimated. Thus, there may exist functionally relevant differences between the average laminar interconnections described here and the specific laminar microcircuits formed within these average patterns. A further dimension that is missing from many descriptions of local microcircuitry is an estimation of connection strength. However, with current technology, structural measures of strength, such as the frequency of connections from one cell type onto another or the number of involved synapses and their morphology, can only be obtained by arduous manual labor. Moreover, the translation of structural into functional strength, as expressed by the amplitude of evoked postsynaptic currents, is opaque: number, size, morphology and position of synapses matter, as do numerous molecular mechanisms regulating synaptic function at both the pre- and postsynaptic site. In addition, the impact of evoked currents on postsynaptic cell function depends on many further factors. All these aspects are not static, but can potentially change on short time scales (Squire et al., 2008; Buonomano and Maass, 2009; Dityatev et al., 2010; Eroglu and Barres, 2010; Silver, 2010; Ribrault et al., 2011; Arnsten et al., 2012; Camiré and Topolnik, 2012; Caroni et al., 2012; Cortés-Mendoza et al., 2013; Dallérac et al., 2013; Vitureira and Goda, 2013; Chevaleyre and Piskorowski, 2014).

Although the proposed intrinsic circuitry for agranular cortex is still speculative, the issue we address remains crucial (Marcus et al., 2014). There has to be variation in intrinsic circuitry across the cerebral cortex, because the composition of the cortex is not uniform, but highly variable on a number of dimensions. We are convinced that a better understanding of the intrinsic cortical circuitry is essential for an improved comprehension of its physiology, and has to take into account differences in the cortical structural substrate. We hope that we have provided a starting point for discussion which will lead to the synthetization of new insights from available data or further experimental efforts to elucidate circuitry outside of striate cortex, taking structural variation into consideration.

## Conflict of interest statement

The authors declare that the research was conducted in the absence of any commercial or financial relationships that could be construed as a potential conflict of interest.
